# Prevalence of resistance to second-line tuberculosis drug among multidrug-resistant tuberculosis patients in Viet Nam, 2011

**DOI:** 10.5365/WPSAR.2016.7.2.002

**Published:** 2016-06-08

**Authors:** Hoa Binh Nguyen, Nhung Viet Nguyen, Huong Thi Giang Tran, Hai Viet Nguyen, Quyen Thi Tu Bui

**Affiliations:** a National Tuberculosis Control Programme Viet Nam, Hanoi, Viet Nam.; b Centre for Operational Research, International Union Against Tuberculosis and Lung Disease, Paris, France.; c Viet Nam Association for Tuberculosis and Lung Disease, Hanoi, Viet Nam.; d Ministry of Health, Department of International Cooperation, Viet Nam.; e Hanoi Medical University, Hanoi, Viet Nam.; f Hanoi School of Public Health, Hanoi, Viet Nam.

## Abstract

**Introduction:**

Extensively drug-resistant tuberculosis (XDR-TB) represents an emerging public health problem worldwide. According to the World Health Organization, an estimated 9.7% of multidrug-resistant TB (MDR-TB) cases are defined as XDR-TB globally. The objective of this study was to determine the prevalence of drug resistance to second-line TB drugs among MDR-TB cases detected in the Fourth National Anti-Tuberculosis Drug Resistance Survey in Viet Nam.

**Methods:**

Eighty clusters of TB cases were selected using a probability-proportion-to-size approach. To identify MDR-TB cases, drug susceptibility testing (DST) was performed for the four major first-line TB drugs. DST of second-line drugs (ofloxacin, amikacin, kanamycin, capreomycin) was performed on isolates from MDR-TB cases to identify pre-XDR and XDR cases.

**Results:**

A total of 1629 smear-positive TB cases were eligible for culture and DST. Of those, DST results for first-line drugs were available for 1312 cases, and 91 (6.9%) had MDR-TB. Second-line DST results were available for 84 of these cases. Of those, 15 cases (17.9%) had ofloxacin resistance and 6.0% were resistant to kanamycin and capreomycin. Five MDR-TB cases (6.0%) met the criteria of XDR-TB.

**Conclusion:**

This survey provides the first estimates of the proportion of XDR-TB among MDR-TB cases in Viet Nam and provides important information for local policies regarding second-line DST. Local policies and programmes that are geared towards TB prevention, early diagnosis and treatment with effective regimens are of high importance.

## Introduction

Tuberculosis (TB) is a leading cause of death in communicable diseases. According to the World Health Organization (WHO), an estimated 9.6 million people developed TB and 1.5 million died from the disease worldwide in 2014. (*1*) Viet Nam ranks 12th among the 22 high-burden TB countries. (*1*) In 2007, the first nationwide TB prevalence survey in Viet Nam showed a prevalence of bacteriological-positive TB of 307 per 100 000 adult population (15 years or older) and a prevalence of smear-positive TB of 197 per 100 000 adult population. (*2*)

Resistance to anti-TB drugs is a public health threat to controlling TB worldwide. (*1*, *3*, *4*) Globally, the proportion of cases with multidrug-resistant TB (MDR-TB) (*4*) is 3.3% among new TB cases and 20% among previously treated TB cases, accounting for about 480 000 people in 2014. (*1*) Viet Nam is among the 27 high MDR-TB burden countries; the estimated proportion of new MDR-TB cases in Viet Nam was 4.0%, and the estimated proportion of MDR-TB among re-treatment cases was 23% in 2014. (*1*, *5*) Based on the Fourth National Anti-Tuberculosis Drug Resistance Survey in Viet Nam and the WHO Global Tuberculosis Report 2015, there were an estimated 5100 cases with MDR-TB among notified TB cases in Viet Nam. (*1*, *5*)

In 2009, the Viet Nam National Tuberculosis Programme (NTP) implemented programmatic management of drug-resistant TB (PMDT) to diagnose and provide treatment with second-line TB drugs (SLDs) for MDR-TB under programme condition. The PMDT has been integrated into the general national TB control programme with additional financial and technical support to manage MDR-TB cases. The number of MDR-TB cases detected and enrolled for MDR treatment increased from 101 in 2009 to 2131 in 2015. These cases account for 42% of the estimated 5100 MDR-TB cases in Viet Nam, which is much higher than the proportion (26%) in the global estimation. (*1*) However, this achievement still needs to be improved to achieve the national target of MDR-TB case enrolment for treatment.

Extensively drug-resistant TB (XDR-TB) represents an emerging public health problem worldwide. (*1*, *3*) According to WHO, an estimated 9.7% of global MDR-TB cases had XDR-TB. (*1*) The emergence and spread of MDR and XDR-TB is a big challenge for TB control activities as treatment of MDR and XDR-TB cases is more expensive, has more adverse drug reactions, is less successful and has higher death rates than other types of TB. (*1*, *3*, *4*, *6*)

In 2011, the Viet Nam NTP conducted the Fourth National Anti-Tuberculosis Drug Resistance Survey in Viet Nam to assess the burden of TB drugs resistance in the country. (*5*) In this study, we performed drug susceptibility testing (DST) for first-line TB drugs to estimate the prevalence of MDR-TB; we also conducted DST for SLDs (ofloxacin, amikacin, kanamycin and capreomycin). We aimed to determine the prevalence of XDR-TB among all MDR-TB cases detected in the Fourth National Anti-tuberculosis Drug Resistance Survey in Viet Nam.

## Methods

### Study subjects, sample size and sampling

The full details of the study design, sampling strategy, sample size calculation and analysis of the Fourth National Anti-Tuberculosis Drug Resistance Survey in Viet Nam are described elsewhere. (*5*) Briefly, it was a cross-sectional survey that used a probability-proportional-to-size (PPS) sampling approach based on notifications of new smear-positive TB cases to assess the prevalence of MDR-TB among TB cases in Viet Nam. This survey was conducted in 80 clusters chosen from all district TB units in the country where TB cases were diagnosed and registered for treatment. Eligible cases were smear-positive TB cases newly registered for treatment (both new and previously treated TB cases) in the selected clusters during the period of recruitment. The required sample size was 1612 new smear-positive TB cases; therefore, each cluster was required to enrol at least 22 new TB cases during the six month period from June to December 2011. All previously treated smear-positive TB cases identified during this period were enrolled in this study.

### Data collection and laboratory procedures

Data collection and laboratory procedures have also been described in detail elsewhere. (*5*) Briefly, TB cases were interviewed face-to-face to collect information on age, sex, region of residence, previous TB treatment history, symptoms and HIV status. Two sputum samples were collected from each patient for culture and DST. The samples were transported, within 48 hours after collection, in chilled containers (2–8 °C) to two national/regional laboratories and five provincial laboratories that have the capacity and facilities for TB culture. The specimens were decontaminated using 4% NaOH, and 0.1 mL of the mixture was inoculated onto two Ogawa tubes (according to the modified Petroff’s method). (*7*) The isolates were then sent to two national laboratories for identification and DST, using the proportion method on Loewenstern-Jensen medium for four drugs: Isoniazid (INH), Rifampicin (RMP), Ethambutol (EMB) and Streptomycin (SM) with the following critical concentrations: INH 0.2 µg/ml, RMP 40 µg/ml, EMB 2 µg/ml and SM 4 µg/ml. The proportion method on Loewenstern-Jensen medium was also conducted for DST for all SLDs including ofloxacin (OFX), amikacin (AM), kanamycin (KM) and capreomycin (CM) with the following critical concentrations: OFX 2 µg/ml, KM 30 µg/ml, AM 40 µg/ml, CM 40 µg/ml, according to WHO standard. (*8*)

To ensure the quality of the DST, all samples of MDR-TB cases’ isolates in the two national hospitals were sent for re-testing by crosscheck between two national and regional laboratories following WHO guidelines for surveillance of drug resistance in TB. (*9*) Quality assurance for the culture and DST in this study was provided by a reference laboratory in Adelaide, Australia.

### TB case definitions

We followed WHO-recommended definitions for TB drugs resistance cases: (*4*)

- MDR-TB: TB caused by strains of *Mycobacterium tuberculosis* that are resistant to at least INH and RMP;
- Pre-XDR-TB: TB resistance to INH, RMP, and to one of the three injectable drugs (KM, AM and CM) or to fluoroquinolones (FQs); and
- XDR-TB: MDR-TB plus resistance to a FQ and at least one second-line injectable agent: AM, KM and/or CM.

### Data management and analysis

All data were extracted from the survey database and laboratory database of the Fourth National Anti-Tuberculosis Drug Resistance Survey in Viet Nam. Data analysis was performed using Stata version 12 SE software (Stata Corporation, College Station, Texas, USA). We calculated the numbers and proportions of MDR-TB cases who had resistance to SLDs (based on the four SLDs as described previously). The 95% confidence intervals (CI) were calculated throughout and the level of significance was set at *P* ≤ 0.05.

The study protocol was approved by the Ethical Institutional Review Board of the National Lung Hospital, Viet Nam.

## Results

The survey was conducted in 80 TB clusters from 1 June to 31 December 2011 and enrolled 1840 smear-positive TB cases. Nine clusters were excluded due to a laboratory contamination problem identified after the end of the case enrolment period in one of the seven culture laboratories. In total, 1552 samples that had been cultured were included in the analysis. Of those, the total number of people diagnosed with smear- and culture-positive TB was 1341; DST results were available for 1312 cases. Of those, 91 MDR-TB cases were reported, including 46 MDR-TB among 1105 new TB cases (4.2%, 95% CI: 2.5–5.4) and 45 MDR-TB among 195 previously treated TB cases (23.1%; 95% CI: 16.7–29.9). **Table 1** shows the demographic characteristics of the 91 MDR-TB cases. The median age of these cases was 46.2 years (range 20–80 years); 27.5% were in the 20–34 year age group. Of the 91 MDR-TB cases, 78.0% were male and 65.9% were residents of the southern region in Viet Nam.

**Table 1:** Characteristics of MDR-TB cases, the Fourth National Anti-Tuberculosis Drug Resistance Survey in Viet Nam, 2011 (*n* = 91)

Characteristics	*n*	%
*Sex*		
Male	71	78.0
Female	20	22.0
*Age group (years)*		
20–34	25	27.5
35–44	14	15.4
45–54	25	27.5
55–64	17	18.7
65–80	10	11.0
*Region of residence in Viet Nam*		
North	16	17.6
Central	15	16.5
South	60	65.9
*Tuberculosis history*		
No TB history	46	50.5
Previous TB	45	49.5

TB, tuberculosis.

### Patterns of resistance to the first-line anti-TB drugs

By definition, all of the 91 MDR-TB cases were resistant to INH and RMP (**Table 2**). In addition, 50 MDR-TB cases (54.9%; 95% CI: 44.5–65.4) were resistant to EMB, 84 (92.3%; 95% CI: 86.7–97.9) were resistant to SM and 47 (51.6%; 95% CI: 41.2–62.1) were resistant to both EMB and SM. Of the 46 new MDR-TB cases, 29 (63.0%; 95% CI: 48.6–77.5) were resistant to EMB, 44 (95.7%; 95% CI: 89.5–100.0) were resistant to SM and 27 (58.7%; 95% CI: 43.9–73.5) were resistant to both EMB and SM. Of 45 re-treated cases, 21 (46.7%; 95% CI: 31.5–61.8) were resistant to EMB, 40 (88.9%; 95% CI: 79.3–98.4) were resistant to SM and 20 (44.4%; 95% CI: 29.3–59.5) were resistant to both EMB and SM.

**Table 2:** Pattern of resistance to anti-TB drugs in MDR-TB cases, the Fourth National Anti-Tuberculosis Drug Resistance Survey in Viet Nam, 2011

	Total		New cases		Previously treated cases	
	Number tested	Resistance *n* (%, 95% CI)	Number tested	Resistance *n* (%, 95% CI)	Number tested	Resistance *n* (%, 95% CI)
*First-line drug*						
Ethambutol (EMB)	91	50 (54.9; 44.5–65.4)	46	29 (63.0; 48.6–77.5)	45	21 (46.7; 31.5–61.8)
Streptomycin (SM)	91	84 (92.3; 86.7–97.9)	46	44 (95.7; 89.5–100)	45	40 (88.9; 79.3–98.4)
Isoniazid (INH)	91	91 (100)	46	46 (100)	45	45 (100)
Rifampicin (RMP)	91	91 (100)	46	46 (100)	45	45(100)
*Second-line drug*						
Amikacin (AM)	84	1 (1.2; 0–3.6)	41	0 (0)	43	1 (2.3; 0–7.0)
Capreomycin (CM)	84	5 (6.0; 0.8–11.1)	41	2 (4.9; 0–11.8)	43	3 (7.0; 0–14.9)
Kanamycin (KM)	84	5 (6.0; 0.8–11.1)	41	2 (4.9; 0–11.8)	43	3 (7.0; 0–14.9)
Ofloxacin (OFX)	84	15 (17.9; 9.4–26.2)	41	8 (19.5; 6.8–32.1)	43	7 (16.3; 4.7–27.8)
Pre-XDR-TB	84	15 (17.9; 9.4–26.2)	41	8 (19.5; 6.8–32.1)	43	7 (16.3; 4.7–27.8)
XDR-TB	84	5 (6.0; 0.8–11.1)	41	2 (4.9; 0–11.8)	43	3 (7.0; 0–14.9)
AM+CM+KM+OFX	84	1 (1.2; 0–3.6)	41	0 (0)	43	1 (2.3; 0–7.0)
CM+KM+OFX	84	5 (6.0; 0.8–11.1)	41	2 (4.9; 0–11.8)	43	3 (7.0; 0–14.9)

CI, confidence interval; TB, tuberculosis; and XDR-TB, extensively drug-resistant tuberculosis.

### Patterns of resistance to the second-line anti-TB drugs

Of the 91 MDR-TB cases, second-line DST results were available for 84 (92.3%) of them (**Table 2**). The overall proportion of pre-XDR was 17.9% (95% CI: 9.4–26.2). The proportion of pre-XDR in new cases (*n* = 41) was 19.5%(95% CI: 6.8–32.1) and 16.3% (95% CI: 4.7–27.8) in previously treated cases (*n* = 43). A further five cases, 6.0% (95% CI: 0.8–11.1) were classified as XDR-TB, including two new (4.9%, 95% CI: 0–11.8) and three previously treated cases (7.0%, 95% CI: 0–14.9).

**Table 3** presents the characteristics of the five XDR-TB cases. All of them were male and were HIV sero-negative. The median age of XDR-TB cases was 48 years with the youngest aged 23 years and the oldest 63 years old. The XDR-MTB isolates originated from the central (*n* = 2) and southern (*n* = 3) regions of Viet Nam.

**Table 3:** Characteristics of XDR-TB cases detected in the Fourth National Anti-Tuberculosis Drug Resistance Survey in Viet Nam, 2011

Case	Age	Sex	Region of residence	TB previously treated	HIV status	Pattern of drug resistance for FLDs	Pattern for drug resistance of SLDs
1	23	Male	Central	No	Negative	H; R; S	CM; KM; OFL
2	53	Male	Central	Yes	Negative	E; H; R; S	CM; KM; OFL
3	63	Male	South	Yes	Negative	E; H; R; S	CM; KM; OFL
4	46	Male	South	No	Negative	E; H; R; S	CM; KM; OFL
5	48	Male	South	Yes	Negative	E; H; R; S	AM; CM; KM; OFL

AM, Amikacin; CM, Capreomycin; E, Ethambutol; FLDs, first-line TB drugs; H, Isonoazid; KM, Kanamycin; OFL, Ofloxacin; R, Rifampicin; SLDs, second-line TB drugs; S, Streptomycin; and TB, tuberculosis.

## Discussion

We observed high levels of resistance to SLDs among MDR-TB cases. Our findings support data presented by other studies. (*4*, *6*, *10*-*14*) The overall proportion of XDR-TB among MDR-TB cases in this study was still lower than the global average estimate. (*1*) Whereas the proportion of XDR-TB among MDR-TB cases elsewhere varied from 5% to 21%. (*4*, *6*, *10*-*14*) However, James et al. reported a very high percentage of XDR-TB among MDR-TB cases (60%) in India. (*15*) This could be due to referral bias because that study was conducted among the cases referred to the tertiary care hospital.

We believe the main reason for XDR-TB is indiscriminate use of antibiotics that are also used as second-line anti-TB drugs. (FQs and others second-line drugs were available in pharmacies in high TB burden countries.) (*16*) It is also due to inadequate treatment by health staff and low compliance to full therapy by TB cases. In Viet Nam, of 1380 MDR-TB cases enrolled for treatment during 2010–2012, 372 (27%) were unable to complete treatment. (This proportion varied in different parts of Viet Nam.) Loss-to-follow-up was the main unfavourable treatment outcome (13%) and may be due to the long treatment period. (*17*)

MDR-TB and XDR-TB are indicators of TB control failures; they emerge due to several reasons: (1) provider may prescribe insufficient drug regiments for TB cases; (2) TB cases may not adhere to an appropriate regimen; (3) drugs may be of poor quality; and (4) there is transmission of MDR-TB and XDR-TB in the community. In Viet Nam, anti-TB drugs (both first-line and second-line drugs) are available without a medical prescription. About half of the private pharmacies were willing to dispense drugs either to TB cases or to those who asked for TB medication. (*18*) We found a high prevalence of resistance to OFX (9.4–26.2%), similar to other studies. (*10*, *13*, *14*, *19*, *20*) This may be due to the fact that TB cases who received treatment may have used OFX before. The high prevalence of pre-XDR TB cases might imply the inappropriate usage of drugs, especially FQs including OFX. This drug is the most commonly prescribed antibiotic for respiratory tract infections as well as other bacterial infections, and in some cases it is available in local drug stores in Viet Nam without presenting a prescription. (*16*) Easy access and inappropriate use of these drugs increase the risk for drug-resistant TB emergence.

In this study, all TB cases who were resistant to KM were also resistant to CM, consistent with other studies. (*21*, *22*) CM is not widely available as it is expensive, and is not commonly used in Viet Nam. Maus et al. reported the cross-resistance between KM and CM in a study in the United States of America. (*21*) The study describes isolates resistant to CM and LM caused by mutations in the rrs gene. The isolates recovered from TB cases treated with KM were resistant to KM and CM, and the resistance of the strains to CM varied with the level of KM resistance. (*21*) Further investigation of the rrs gene for MDR-TB cases is needed to confirm the cross-resistance to KM and CM in Viet Nam.

There are limitations in this study. First, the clusters were selected based on the notification of new smear-positive TB cases in 2003 but not on more recent data. However, case distribution for TB notifications remained fairly stable from 2003 to 2011. (*5*) Second, this study did not include the private health sector, therefore we could not estimate the burden of MDR-TB in the private sector. Nevertheless, in spite of these shortcomings, samples in this study generally represent all of Viet Nam.

## Conclusions

This survey provides the first estimates of the proportion of XDR-TB among MDR-TB cases in Viet Nam. The results provide important information for clinicians and local policy-makers as well as international health agencies regarding the conduct of second-line DST. Local TB policies and programmes that are geared towards prevention, early diagnosis and treatment with effective regimens are of high importance.

## References

[1] Global tuberculosis report 2015. Geneva World Health Organization 2015 http://apps.who.int/iris/bitstream/10665/191102/1/9789241565059_eng.pdf?ua=1 accessed 16 May 2016

[2] Hoa NB, Sy DN, Nhung NV, Tiemersma EW, Borgdorff MW, Cobelens FG. National survey of tuberculosis prevalence in Viet Nam. Bull World Health Organ. 2010 4;88(4):273–80. 10.2471/BLT.09.067801 20431791 PMC2855599

[3] Anti-tuberculosis drug resistance in the world, Report No. 4: The WHO/IUATLD global project on anti-tuberculosis drug resistance surveillance.Geneva World Health Organization 2008 http://apps.who.int/iris/bitstream/10665/43889/1/WHO_HTM_TB_2008.394_eng.pdf?ua=1&ua=1 accessed 16 May 2016

[4] (2010). Multidrug and extensively drug-resistant TB (M/XDR-TB): 2010 global report on surveillance and response..

[5] Nhung NV, Hoa NB, Sy DN, Hennig CM, Dean AS. The fourth national anti-tuberculosis drug resistance survey in Viet Nam. Int J Tuberc Lung Dis. 2015 6;19(6):670–5. 10.5588/ijtld.14.0785 25946357

[6] Poudel A, Maharjan B, Nakajima C, Fukushima Y, Pandey BD, Beneke A, et al. Characterization of extensively drug-resistant Mycobacterium tuberculosis in Nepal. Tuberculosis (Edinb). 2013 1;93(1):84–8. 10.1016/j.tube.2012.10.007 23146281

[7] Guidelines on Standard Operating Procedures for Laboratory Diagnosis of HIV-Opportunistic Infections.New Delhi World Health Organization Regional Office for South-East Asia 2001 http://apps.searo.who.int/PDS_DOCS/B0189.pdf accessed 16 May 2016

[8] Policy guidance on drug-susceptibility testing (DST) of second-line antituberculosis drugs.Geneva World Health Organization 2008 http://apps.who.int/iris/bitstream/10665/70500/1/WHO_HTM_TB_2008.392_eng.pdf accessed 16 May 2016 26290924

[9] (2009). Guidelines for surveillance of drug resistance in tuberculosis. Fourth edition.

[10] Paramasivan CN, Rehman F, Wares F, Sundar Mohan N, Sundar S, Devi S, et al. First- and second-line drug resistance patterns among previously treated tuberculosis patients in India. Int J Tuberc Lung Dis. 2010 2;14(2):243–6. 20074419

[11] Migliori GB, D’Arcy Richardson M, Sotgiu G, Lange C. Multidrug-resistant and extensively drug-resistant tuberculosis in the West. Europe and United States: epidemiology, surveillance, and control. Clin Chest Med. 2009 12;30(4):637–65, vii. [vii.] 10.1016/j.ccm.2009.08.015 19925959

[12] Li X, Wang H, Jing H, Wang Y, Yu C, Wang J, et al. Population-based surveillance of extensively drug-resistant tuberculosis in Shandong Province, China. Int J Tuberc Lung Dis. 2012 5;16(5):612–4. 22410186 10.5588/ijtld.11.0507

[13] Hu Y, Hoffner S, Wu L, Zhao Q, Jiang W, Xu B. Prevalence and genetic characterization of second-line drug-resistant and extensively drug-resistant Mycobacterium tuberculosis in Rural China. Antimicrob Agents Chemother. 2013 8;57(8):3857–63. 10.1128/AAC.00102-13 23733477 PMC3719720

[14] Punga VV, Jakubowiak WM, Danilova ID, Somova TR, Volchenkov GV, Kazionnyy BY, et al. Prevalence of extensively drug-resistant tuberculosis in Vladimir and Orel regions, Russia. Int J Tuberc Lung Dis. 2009 10;13(10):1309–12. 19793439

[15] James P, Gupta R, Christopher DJ, Thankagunam B, Veeraraghavan B. MDR- and XDR-TB among suspected drug-resistant TB patients in a tertiary care hospital in India. Clin Respir J. 2011 1;5(1):19–25. 10.1111/j.1752-699X.2009.00184.x 21159137

[16] Wells WA et al Size and usage patterns of private TB drug markets in the high burden countries. PLoS One 2011 6(5):e18964 10.1371/journal.pone.0018964 21573227 PMC3087727

[17] Phuong NTM et al.Management and treatment outcomes of patients enrolled in MDR-TB treatment in Viet Nam. Public Health Action 2016 6(1):25 31. 10.5588/pha.15.0068 27051608 PMC4809724

[18] Vu DH, van Rein N, Cobelens FG, Nguyen TT, Le VH, Brouwers JR. Suspected tuberculosis case detection and referral in private pharmacies in Viet Nam. Int J Tuberc Lung Dis. 2012 12;16(12):1625–9. 10.5588/ijtld.12.0295 23131260

[19] Iqbal R, et al. The First and Second Line Anti TB Drug Resistance Pattern in Lahore. Pakistan. J Med Res. 2012;51(1)

[20] Agrawal D et al.Increasing incidence of fluoroquinolone-resistant Mycobacterium tuberculosis in Mumbai, India. International Journal of Tuberculosis and Lung Diseases 2009 13(1):79 83. 19105883

[21] Maus CE, Plikaytis BB, Shinnick TM. Molecular analysis of cross-resistance to capreomycin, kanamycin, amikacin, and viomycin in Mycobacterium tuberculosis. Antimicrob Agents Chemother. 2005 8;49(8):3192–7. 10.1128/AAC.49.8.3192-3197.2005 16048924 PMC1196259

[22] Jugheli L, Rigouts L, Shamputa IC, Bram de Rijk W, Portaels F. High levels of resistance to second-line anti-tuberculosis drugs among prisoners with pulmonary tuberculosis in Georgia. Int J Tuberc Lung Dis. 2008 5;12(5):561–6. 18419893

